# CT-like MR images to assess changes after radiotherapy for bone metastasis: a case report

**DOI:** 10.1093/bjrcr/uaaf018

**Published:** 2025-03-24

**Authors:** Osamu Tanaka, Takuya Taniguchi, Takuji Kiryu, Ryoshu Maejima, Chiyoko Makita, Masayuki Matsuo

**Affiliations:** Department of Radiation Oncology, Asahi University Hospital, Gifu 500-8523, Japan; Department of Radiation Oncology, Asahi University Hospital, Gifu 500-8523, Japan; Department of Radiology, Asahi University Hospital, Gifu 500-8523, Japan; Department of Radiation Oncology, Asahi University Hospital, Gifu 500-8523, Japan; Department of Radiation Oncology, Gifu University Hospital, Gifu 501-1194, Japan; Department of Radiation Oncology, Gifu University Hospital, Gifu 501-1194, Japan

**Keywords:** MRI, radiotherapy, bone metastasis

## Abstract

Setting the echo time to zero allows for the acquisition of bone images that were otherwise difficult to obtain with conventional MRI and clear visualization of CT-like MR images. This technique is mainly useful for detecting compression fractures; however, studies examining bone tumours have been lacking. Furthermore, no reports to date have investigated the usefulness of MRI for evaluating images before and after radiotherapy (RT) for bone tumours. Therefore, plain CT and MRI (T1/T2-weighted image and CT-like MRI) were performed under the same conditions before and after radiation therapy (RT) and examined the obtained images. An 86-year-old man received RT (30 Gy/3 fraction) for painful lumbar metastasis from prostate cancer. At 2 months after RT, no changes in T2-weighted images and plain CT scans were noted, but CT-like MRI showed an increase in the signal inside the bone metastasis. Examining how the images change over time is imperative given the difficulty of predicting the duration of the pain relief effects of RT for bone metastases. Therefore, the current case report explored whether combining various modalities, such as CT and MRI, could predict prognosis. We highlight the importance of investigating whether signal changes are correlated with pain symptoms and whether MRI can be a predictor.

## Introduction

We herein report a novel MRI technique termed FRACTURE (alternatively called zero echo time [ZTE] and ultrashort echo time [UTE]), which provides superior cortical and trabecular contrast compared to conventional MRI sequences, possesses high diagnostic value, and delivers clinically relevant information.^1–5^ Currently, FRACTURE has been frequently utilized as a complement to traditional musculoskeletal MRI sequences of various conditions, such as anterior shoulder instability, complex fractures, lumbar spondylolysis, and erosions associated with inflammatory arthritis.^2^^,^^3^^,^^5^ Preliminary case subset analyses that directly compared FRACTURE with CT and conventional radiography reported high concordance.^2–5^

Although studies have confirmed the usefulness of FRACTURE in benign diseases, to the best of our knowledge, no study has reported on its usefulness in cancer. Therefore, the current case report examined the changes in CT/MRI (FRACTURE) images before and after radiation therapy (RT) for bone metastasis of cancer.

## Clinical presentation

An 86-year-old man initially started treatment with bicalutamide and leuprorelin acetate for locally advanced prostate cancer. Risk stratification was performed according to the D’Amico classification, revealing that the patient was at high risk (T3aN1M1b), which steadily decreased his prostate-specific antigen levels. However, 6 months into his treatment, he was referred to the Department of Radiation Oncology owing to an increase in the metastasis to the fifth lumbar vertebra upon undergoing lumbar spine examination for disease exacerbation. Before RT, he received only celecoxib (200 mg/day); however, after 1 month, his pain disappeared, and he no longer needed painkillers. At presentation, the Gleason score and PSA were 5 + 5 and 3.85 ng/mL, respectively. Six months after RT, the prostate-specific antigen (PSA) had dropped to 0.65 ng/mL. Given that no recurrence of pain was noted during the 6 months of observation, he was subsequently transferred to his family doctor after completing the treatment for oligometastasis. Written informed consent was obtained from the patient for publication, including accompanying images.

### Imaging findings

**Figure 1.**
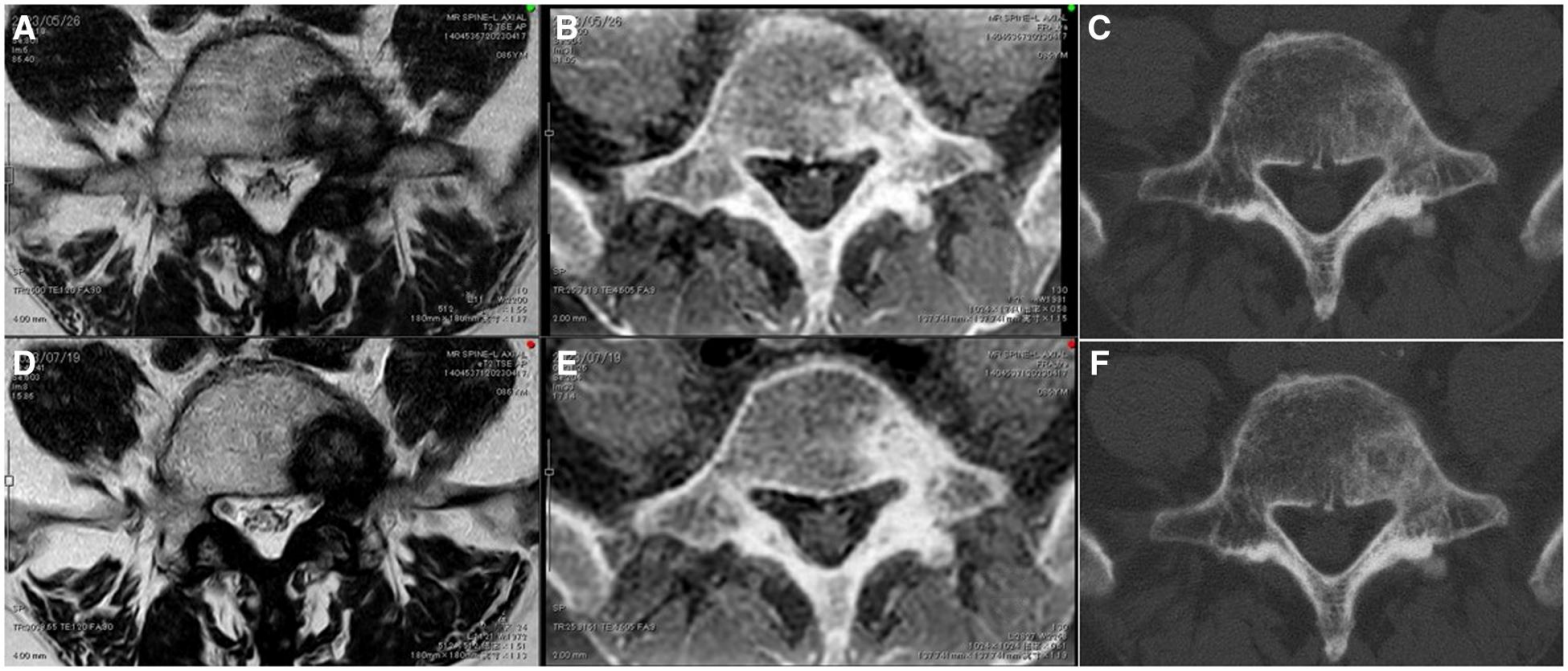
Comparison of images before and after radiation therapy. Panels A and D show T2-weighted images; panels B and E show CT-like magnetic resonance images, and panels C and F show plain CT images. Panels A, B, and C show images before radiotherapy for bone metastasis, whereas panels D, E, and F show images after radiotherapy. Panels B and E show an increase in the area of bone sclerosis before and after radiotherapy.

Furthermore, MRI and CT revealed metastasis to the fifth lumbar vertebra (Figure 1A-C). The FRACTURE imaging sequence is provided in Table 1. T1-weighted imaging (T1WI) usually provides a clear border between the tumour and normal tissue. However, we used both T1WI and T2-weighted imaging (T2WI) to clearly observe the boundary between the spinal canal and tumour. Simultaneously, osteosclerosis was clearly depicted in the FRACTURE image. However, the margin of the tumour lesion was unclear on plain CT. CT images were acquired using the following settings: 16-row detector CT; thickness, 1.25 mm; field of view, 40 × 40 cm^2^; 120 kV; and 460 mA.

**Table 1.** Sequence of FRACTURE.

	T2-weighted image	FRACTURE
TE (echo time), ms	120	4.6/9.2/13.8/18.4
TR (repetition time), ms	2500	26
Flip angle	90	9
Field of view, mm	180/180/13	300/300/90
Acquisition voxel, mm	0.51/0.77/4.00	1.19/1.20/1.20
Reconstruction voxel, mm	0.35/0.35/0.35	0.63/0.63/0.60
Parallel imaging	No	No

### Radiotherapy settings

The Elekta Synergy linear accelerator was used with coplanar volume modulated arc therapy [Elekta AB (Publ), Box 7593 SE-103 93, Stockholm, Sweden]. The tumour was considered to have been controlled in the prostate and was in the state of oligometastasis.

**Figure 2.**
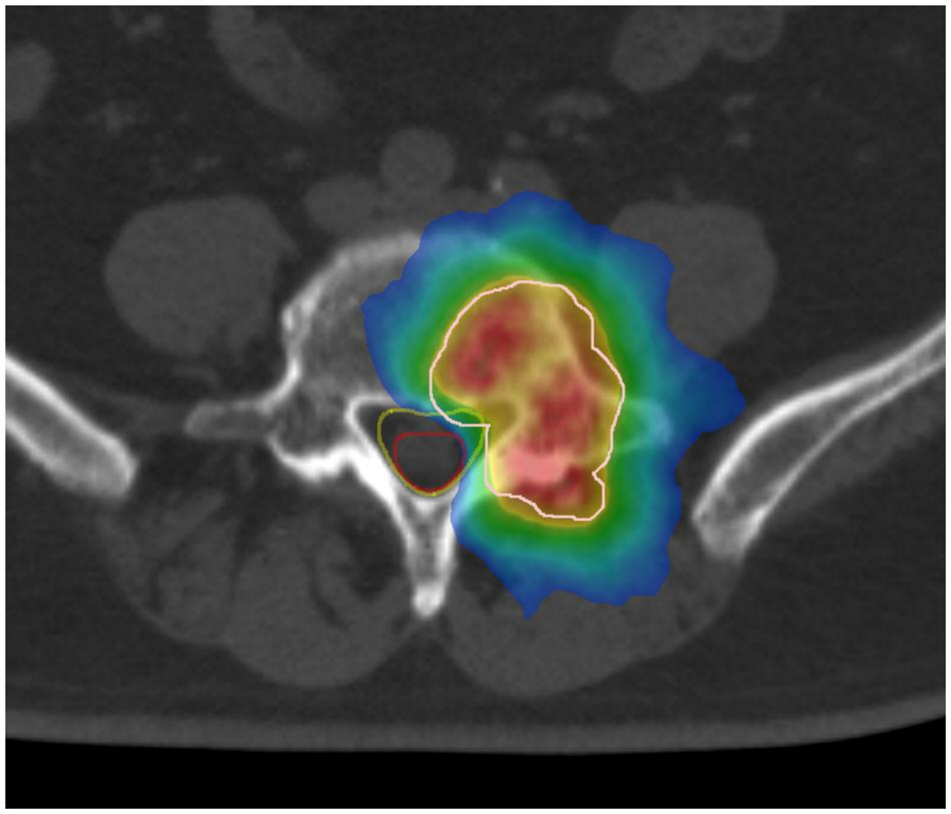
Dose colour wash of the radiotherapy. White line, PTV; red line, cauda equina; yellow line, spinal canal. (Red area) The prescribed dose is delivered to 105% of the PTV. (Yellow area) The prescribed dose is delivered to 100% of the PTV. (Green area) The prescribed dose is delivered to 95% of the PTV. (Blue area) The prescribed dose is delivered to 50% of the PTV.

Gross target volume (GTV) was defined as the range in which the tumour could be recognized through MRI with vertebral body spinal stereotactic body RT (30 Gy/3 fractions). As previously reported, the organ at risk (OAR) was the cauda equina nerve and not the spinal canal. The clinical target volume (CTV) sets the GTV with a 2-mm margin, whereas the PTV sets the CTV with a 2-mm margin. Given that the cauda equina nerve is an OAR, the overlap area was cut so that the OAR would not be included in the PTV (Figure 2).

### Outcomes

#### Changes in pain

At the first visit, the patient had a Numerical Rating Scale (NRS) of 4 for lower back pain, but subsequent administration of celecoxib (200 mg/day) kept it at 0. However, an acute exacerbation event occurred, which increased his NRS to 7 despite celecoxib administration (200 mg/day). Thus, we started prescribing morphine hydrochloride hydrate (5 mg, 3-4 times a day) as needed for pain and performed single-fraction 8-Gy RT.

A month after RT, morphine hydrochloride hydrate was no longer necessary, with the patient’s NRS remaining at 2 with the use of celecoxib (200 mg/day). Two months after RT, his NRS decreased to 0 without the use of painkillers, which persisted until 6 months after RT. The patient was then followed up by his family doctor.

#### Changes in imaging

At 2 months after radiotherapy, MRI and CT were performed using the same imaging approach used before treatment (Figure 1D-F). T2WI and CT showed no changes in the tumour relative to that before treatment; however, on FRACTURE, the signal inside the tumour increased, with changes having been observed after RT. Pain disappeared 2 months after RT.

## Discussion

The current case report investigated the usefulness of FRACTURE before and after radiotherapy for bone metastasis. The following data were obtained from our case: (1) FRACTURE can clearly visualize the boundaries between tumours and nearby ligaments and soft tissues as well as T1WI/T2WI^2^^,^^5^; (2) CT can clearly visualize the degree of bone sclerosis of lesions^3^^,^^4^; and (3) changes inside the tumour after RT can be visualized more clearly with FRACTURE than with T1WI/T2WI and CT.

Although MRI is a good imaging modality for the brain and soft tissues, which contain a considerable amount of water, it struggles with visualizing the bones and lung fields, which are mainly visualized using CT. FRACTURE (UTE and ZTE) is an imaging method used to observe tissues that cannot be visualized by extremely shortening the echo time using conventional imaging modalities.

Normal MRI typically uses a TE of several milliseconds; however, tissues with a short T2 value have rapid signal attenuation due to transverse relaxation, which prevents signal acquisition with normal echo time (TE). Using a short TE of <1 ms in UTE allows for the imaging of tissues with a short T value. Ligaments, tendons, menisci, cortical bones, and osteochondral tissues are some examples of tissues with short T2 and T2* values, with some studies reporting the use of UTE for evaluating these organs.^2^

During the depiction of bone metastasis, the extent of tumour spread is recognized much easier with MRI than with CT. Internal changes after treatment can be clearly visualized with MRI; however, these areas will become unclear when the ligaments, surrounding fascia, or tendons come in contact with each other. T1WI/T2WI remains the gold standard for detecting soft tissue structures; however, in the case of metastasis that does not cause extrabone invasion, we believe that FRACTURE may also be useful in guiding T1WI/T2WI.^1^

We would also like to emphasize the high sensitivity of FRACTURE in detecting changes after treatment, underscoring the usefulness of the sequence. Although T1WI/T2WI certainly remains the gold standard, FRACTURE is a versatile imaging modality with various possibilities for use. Thus, our hospital has just begun to study the correlation between clinical findings, such as pain, and various imaging techniques, including T1WI/T2WI and diffusion-weighted image.

### Radiotherapy setting

Three consensus contouring guidelines have been published to guide the delineation of clinical target volumes in the intact spine, postoperative spine, and sacrum.^6–10^ Notably, one study identified 60 deviations (16.7%) from the consensus contouring guidelines in 283 patients with 360 discrete lesions. Deviation from the guidelines has been associated with inferior local control (LC) (1-year LC: 63.0% vs 85.5%, *P* < 0.001).^9^ Given that contouring in the current study did not match the consensus guidelines, the tumour site was contoured as the GTV using CT, MRI, and 18F-fluorodeoxyglucose positron emission tomography (FDG-PET) (image not shown).

The guidelines published by Cox et al. state that the CTV should (1) include abnormal marrow signals indicating microscopic invasion; (2) include bony CTV expansion to account for subclinical spread; and (3) contain GTV.^6^

Herein, although CTV was smaller than the reference guidelines, the treatment goal for pain control was achieved. We considered the patient’s advanced age, selected the minimum radiation field necessary from the quality-of-life viewpoint, and achieved LC. However, further follow-up is needed to evaluate the duration of LC. To maximize the safety and efficacy of RT, an important concept is keeping radiation exposure as low as reasonably achievable. Following this principle, CT-like MRI has been used to accurately identify the tumour location and shape, thereby optimizing the target contouring for radiation. Consequently, pain had been well managed, with no LC issues having been currently noted. However, further long-term follow-up would be necessary to monitor LC in our case.

### Assessment of pain associated with bone metastasis

Our hospital uses the NRS scale for all patients. Accordingly, our patient had an NRS score of 7 before treatment. After RT, his NRS score decreased to 0, a state considered a complete response.

However, future clinical trials on bone metastases would need to follow the content presented in the update of the international consensus on palliative radiotherapy endpoints by Chow et al.^10^ We believe that validated quality-of-life instruments specific to bone metastases, such as the European Organization for Research and Treatment of Cancer QLQ-BM22 and/or the QLQ-C15-PAL, should be incorporated into the management of affected patients.

## Conclusions

FRACTURE allows for new morphological and quantitative evaluations of diseases by imaging areas that could not be observed through conventional imaging methods. Aside from its ability to perform imaging under free breathing given its ability to withstand movement, various techniques for shortening the imaging time, particularly by devising sampling and reconstruction methods, are being researched. Moreover, further technological development can be expected in the future, which is projected to reduce the burden on patients.^1^

We believe that the numerous MRI techniques and their application technology will certainly contribute to the development of new imaging methods in the near future and that the value of diagnostic imaging using MRI will further increase with time.

## Learning points

- To date, CT has been unable to visualize soft tissues surrounding bones or the internal structure of bones. Although MRI provides a high image resolution, it struggles to visualize minute fractures. Through CT-like MRI, visualizing changes inside bones, such as compression fractures, is now possible.
- This has been the first ever report comparing CT-like MRI imaging before and after radiotherapy for bone metastases. Notably, we were able to detect changes in internal structures more quickly with CT-like MRI than with plain CT. In the future, we hope that research will investigate whether these bone changes, clinical findings, and treatment response rates can be predictive factors. The use of FRACTURE may allow for the observation of changes in the cortical bone, such as remineralization caused by radiation therapy, and in the bone marrow that has been replaced by cancer.
- Any correlations between bone changes on early MRI scans before and after RT could be predicted early after palliative RT, which would be beneficial for the affected patients. Considering that patients place considerable importance on questions such as “When will the pain go away?” and “How long will the pain relief last?,” addressing these questions through non-invasive imaging examinations could improve the quality of life of the affected patients.
